# Recalculating the Surface Ocean CO_2_ Atlas (SOCAT) to a sea surface temperature climate data record

**DOI:** 10.1038/s41597-026-07532-5

**Published:** 2026-06-09

**Authors:** Daniel J. Ford, Jamie D. Shutler, Thomas Holding, Richard P. Sims, Ian Ashton, David K. Woolf, Lonneke Goddijn-Murphy, Dorothee C. E. Bakker, Christopher Sabine, Christopher J. Merchant, Owen Embury, Yuanxu Dong, Rik Wanninkhof

**Affiliations:** 1 Centre for Geography and Environmental Sciences (CGES), University of Exeter, Penryn, UK; 2 College of Engineering Mathematics and Physical Sciences, University of Exeter, Penryn, UK; 3 International Centre for Island Technology, Heriot-Watt University, Stromness, Orkney UK; 4 Environmental Research Institute, University of the Highlands and Islands, Thurso, UK; 5 Centre for Ocean and Atmospheric Sciences, School of Environmental Sciences, University of East Anglia, Norwich, UK; 6 Department of Oceanography, University of Hawaiʻi at Mānoa, 1000 Pope Rd., Honolulu, Hawaii 96822 USA; 7 Department of Meteorology, University of Reading, Reading, UK; 8 National Centre for Earth Observation, University of Reading, Reading, UK; 9 Marine Biogeochemistry Research Division, GEOMAR Helmholtz Centre for Ocean Research Kiel, Kiel, 24148 Germany; 10 Institute of Environmental Physics, Heidelberg University, Im Neuenheimer Feld 229, 69120 Heidelberg, Germany; 11 NOAA/OAR Atlantic Oceanographic and Meteorological Laboratory, Miami, Florida 33149 USA; 12 Present Address: BirthRites Lise Meitner Research Group, Max Planck Institute for Evolutionary Anthropology, Leipzig, Germany; 13 Present Address: Government of Jersey Marine Resources, Natural Environment, Howard Davis Farm, La Route de la Trinitie, Trinity, JE3 5JP Jersey

## Abstract

The Surface Ocean CO_2_ Atlas (SOCAT) is a global scientific community effort to collate and provide additional quality control and standardisation for surface ocean carbon dioxide (CO_2_) data. Each year the international marine carbon community submit any new measurements collected on research vessels, ships of opportunity, moorings, uncrewed surface vehicles and sailing yachts for inclusion in the annual update of the SOCAT database. The data synthesis effort, which published its first data product in 2011, includes a variety of systems, sampling strategies, maintenance cycles and instrument calibrations. Each in-water CO_2_ gas measurement is paired, and linked, with a sea surface temperature (SST) measurement. However, the differences in measurement systems means that data pairs from different platforms are representative of differing depths in the ocean, whilst SST measurements can suffer from warming within the observation platform. These complexities can limit the accuracy and precision of any atmosphere-ocean CO_2_ assessments that use the SOCAT products. Here the SOCATv2025 database with an estimated uncertainty in the fugacity of CO_2_ in seawater (*f*CO_2 (sw)_) of less than 5 µatm is recalculated to a reference temperature at a consistent depth of 0.2 m using the European Space Agency (ESA) Climate Change Initiative (CCI) SST climate data record. This recalculation process of the *f*CO_2_ values does not assume isochemical conditions and so temperature driven carbonate speciation is captured. The data pairing is maintained so the resulting dataset is well suited for the analysis of atmosphere-ocean CO_2_ exchange. The synthesis cruise data and gridded data products, that include both the original and recalculated data, are provided and consistency with the original SOCAT data products and format is confirmed. The importance of robustly accounting for the observed warm bias is demonstrated as removing this signal by recalculation to a climate data record temperature shows a ~0.4 Pg C yr^−1^ (~12%) increase in the 2024 ocean CO_2_ sink (3.4 Pg C yr^−1^). These recalculated data products are needed for annual carbon assessments therefore these will be routinely provided each year following each annual SOCAT dataset release.

## Background & Summary

The ever-increasing anthropogenic carbon dioxide (CO_2_) emissions force the global oceans to absorb more CO_2_. This annual absorption, or sink, is increasing with time and has reached 3.2 ± 0.4 Pg C yr^−1^ during the period 2015–2024^1^. Consequently the ocean has sequestered ~25% of anthropogenic emissions during this decade^1^. Our ability to routinely quantify the annual ocean CO_2_ sink comes from: (1) global ocean biogeochemical models and (2) statistical multi-parameter regression and machine learning approaches of *in situ* observations of the fugacity of CO_2_ in seawater (*f*CO_2 (sw)_). These assessments require *in situ* observations which, via a large voluntary community effort, are annually collected, quality controlled and compiled into the Surface Ocean CO_2_ Atlas (SOCAT)^2^. The latest release, SOCATv2025^3^ contains 41.4 million *f*CO_2 (sw)_ observations, each with an estimated uncertainty of less than 5 µatm spanning 1957 to 2024, with most of the data between 1990 and 2024 (99.7%). These data provide the basis for the observation-based assessments of *f*CO_2 (sw)_ and air-sea CO_2_ fluxes and are also used for the evaluation of ocean biogeochemical models. The SOCAT dataset is therefore a pivotal dataset for the ocean carbon and climate communities.

Air-sea CO_2_ exchange, or flux, occurs across the air-water interface and the rate is largely controlled within the top ~0.02 m of the ocean^4,5^. But due to the physical limitations and differences in sampling techniques across the ships and other platforms, the *f*CO_2 (sw)_ observations within SOCAT are not representative of the top 0.02 m, and the depth can vary depending on the ship or observational platform. Although SOCAT reports observation depth, this depth does not always reflect the true sampling depth as ballasting, cargo and sea state, for example, can vary the true sampling depth. Consequently the sampling depths within SOCAT are variable (with the average and variance unknown) but will not be representative of the depth directly relevant for air-sea CO_2_ exchange. Alongside these depth-related issues, a warm bias has also been identified within the SOCAT observations^6,7^. This warm bias stems from a multitude of temperature related issues that can affect the seawater temperature measurement (reported as the coincident SST) in SOCAT including: (1) the inadequate calibration of SST sensors, (2) uncorrected warming of the seawater within the ship after the temperature observations are taken (i.e., the SST measurement is generally taken near the intake, whereas the CO_2_ measurement is taken some time later after the water has travelled within the ship).

The presence of the warm bias is well understood, but its magnitude varies depending upon the method used to detect it. Goddijn-Murphy *et al*.^6^ suggested the global mean bias was on order ~0.09 °C between 1991 and 2007 when comparing monthly 1° (i.e 1° latitude by 1° longitude grid) binned SOCAT temperature observations to a satellite temperature dataset that was designed to represent the SST representative of air-sea CO_2_ exchange. Dong *et al*.^7^ indicated the global mean bias was on order ~0.02 °C between 1982 and 2022, when comparing a subset of the monthly 1° binned SOCAT temperature observations to coincident drifting buoys measuring SST (sampling depth ~0.2 m). Dong *et al*.^7^ also showed a latitudinal variability to the magnitude of the warm bias, varying from 0.04 °C at the equator to 0.14 °C at 25°N to −0.05 °C at 35°N and 0.15 °C at 50°N (and a similar variation in the Southern Hemisphere). The *f*CO_2 (sw)_ in the SOCAT dataset can be recalculated or “corrected” to a new temperature dataset allowing for the removal of the warm bias. Careful choice and use of a SST climate data record which is stable with respect to independent observations through time for this recalculation, provides data representative of a constant depth that is directly relevant for air-sea CO_2_ fluxes^4^. The recalculated *f*CO_2 (sw)_ can be used to quantify the impact of this seemingly small warm bias. Watson *et al*.^8^ suggested this warm bias would increase the global ocean CO_2_ sink by 0.4 Pg C yr^−1^ using the Goddijn-Murphy *et al*.^6^ approach to recalculate the SOCAT data, Dong *et al*.^7^ later revised this to an increase of 0.2 Pg C yr^−1^.

The previous assessments of the warm bias, however, have caveats. Both compare monthly 1° binned SOCAT observations to monthly 1° reference data, which inherently introduces both a spatial and temporal mismatch between the differing observations. For example, Goddijn-Murphy *et al*.^6^ use of satellite based observations could be affected by a cool bias within the satellite observations with respect to SST drifter observations^9,10^, that would overestimate the warm bias within the SOCAT temperature observations. Meanwhile, the Dong *et al*.^7^ approach is limited in the ability to capture any drift in this warm bias through time due to the small number of SST drifters coincident to SOCAT observations. These example caveats indicate that an approach that uses the strengths of both observational types will likely produce a more robust evaluation of, and correction for, the warm bias.

Since 2019, a team at the University of Exeter have followed the methods of Goddijn-Murphy *et al*.^6^, and produced a recalculated SOCAT dataset that addresses this warm bias. In early versions, the SOCAT observations were recalculated to the Optimum Interpolated SST data record (OI-SSTv2.0)^11–13^. After 2021, the recalculated dataset^14^ was updated to use OI-SSTv2.1, where the v2.1 SST data addressed an observed cool bias (0.14 °C) in the original OI-SSTv2.0 temperature record^9^. Since 2022, a recalculated version of the SOCAT data using the European Space Agency (ESA) Climate Change Initiative (CCI) SST v2.1^15^ climate data record has been provided alongside the OI-SST referenced version^16,17^.

In this paper and the associated dataset, we consolidate recent efforts to improve the recalculated SOCAT observation dataset, through different lines of evidence. Firstly, the SOCAT observations are recalculated to a daily reference satellite SST dataset at the native spatial resolution of the SST data record from the platform, instead of monthly 1° data. This step reduces the spatial and temporal mismatch between the observational types. Secondly, the reference temperature dataset is updated to the most recent ESA CCI-SSTv3.0^10,18^ which provides daily ~5 km satellite based SST observations at a fixed time of 10:30 h (local solar time) and a depth of 0.2 m. Thirdly, the CCI-SSTv3.0 are corrected for a cool bias (~0.04 °C) that is characterised to exist within the CCI-SST reference satellite SST dataset^19,20^ following recommendations in Dong *et al*.^7^. Finally, the temperature relationship used to transform the *f*CO_2 (sw)_ data to be representative of the CCI-SST temperature is updated (from the previously used quadratic Takahashi *et al*.^21^ relationship) to the more recent advance recommended in Humphreys^22^ which does not assume isochemical conditions.

## Methods

### SOCATv2025 individual cruise synthesis file

The SOCATv2025 dataset was retrieved as a synthesis text file containing individual cruises with an estimated *f*CO_2 (sw)_ uncertainty of less than 5 µatm (containing data Flag as A, B, C or D) from the National Centers for Environmental Information (NCEI)^3^ covering the period 1957 to 2024. The synthesis text file contains the individual *f*CO_2 (sw)_ and coincident SST observations at the seawater intake along with important ancillary information, including the coincident equilibrator temperature and pressure, sea surface salinity (SSS) and air pressure.

We also retrieved the SOCATv2025 Flag E data, which contains individual cruise observations with an estimated *f*CO_2 (sw)_ uncertainty of less than 10 μatm, but greater than 5 μatm. The methods and technical validation will focus on the main SOCATv2025 dataset (i.e observations with estimated *f*CO_2 (sw)_ uncertainty less than 5 μatm), but the recalculation process has also been conducted for this Flag E dataset and are included within the dataset record.

### The CCI-SST climate data record and cool bias correction

The ESA CCI-SSTv3.0 daily Level 4 analysis^10^ is a satellite based SST climate data record that is constructed from both infrared and microwave satellite radiometers. Compared to the previous version of ESA CCI-SST (v2.1), version 3.0 contains improvements to the temporal stability (with fewer timeseries spikes), and the handling of SST retrievals under dust aerosol conditions^10^. The Level 4 analysis uses 22 available radiometer observations that have been consistently recalibrated, and bias adjusted and combines these with an interpolation analysis to produce a daily gap filled dataset at a spatial resolution of 0.05° (~5 km at the equator). These SST have been adjusted to a time of 10:30 h (local time) at a depth of 0.2 m. The time-of-day standardisation avoids aliasing the varying diurnal-cycle sampling from the changing constellation of satellites into the climate record which would manifest as a time-varying SST bias; the CCI-SST record is unique in removing this issue. The interpolation techniques used to produce spatially complete Level 4 products do result in a level of data smoothing which varies spatially, especially when filling across larger gaps. In the case of CCI-SST the Level 4 feature resolution is typically coarser than 15 km. Alongside each SST observation, an SST uncertainty that incorporates the underlying satellite observation and interpolation uncertainties is provided which is representative of the standard deviation of a normal distribution. This approach of characterising the uncertainties has been verified against *in situ* observations^10^. The CCI-SSTv3.0 record was retrieved from Centre for Environmental Data Analysis (CEDA)^18^, and covers the period 1980 to 2024.

The CCI-SST analysis does not ingest *in situ* SST observations from drifters (which are also representative of ~0.2 m depth) or any other platforms. This independence allows *in situ* observations to be routinely used to objectively assess the accuracy and precision of the CCI-SST analysis. The CCI-SST validation report^20^ indicates that, compared to *in situ* SST drifters, the CCI-SST has a relatively stable global cool bias of ~0.04 °C (median) through time. The same validation report showed a small latitudinal variation in the cool bias with a median bias near 0 °C in the subtropics (35°S to 20°S and 20°N to 35°N), and a larger cool bias ~0.1 °C in the high latitude northern hemisphere (above 55°N)^20^. The stability of the CCI-SST record was also independently confirmed by a climate data record intercomparison^19^, which also used *in situ* SST drifter observations. Here, this work is attempting to provide a dataset most representative of the near-surface conditions, therefore before input into the recalculation process this cool bias was removed from the CCI-SST data assuming a globally fixed bias (i.e., 0.04 °C was added to all CCI-SST values). An alternative choice of SST climate data record was the OI-SSTv2.1^9^ but due to temporal stability issues this was less suitable as the reference dataset. This choice and issue are discussed further within the technical validation.

### Matching SOCAT observations to daily reference data

The GitHub version controlled software that followed the method within Goddijn-Murphy *et al*.^6^ was used as the starting point. Within Goddijn-Murphy *et al*.^6^ the satellite reference SST data were linearly interpolated to the locations of the SOCAT observations, where the reference dataset was monthly 1° satellite observations. This software was updated to allow for different spatial resolution reference data to be provided and the ability to flexibly provide the time resolution of the reference data (from daily to calendar month). The daily reference SST and uncertainty data were linearly interpolated in space to the SOCAT observations, providing both SST and SST uncertainty values from the reference data representative for each individual SOCAT observation.

### Recalculation of *f*CO_2 (sw)_ to the reference temperature

The recalculation of the *f*CO_2 (sw)_ observations involves transforming the *f*CO_2 (sw)_ at the reported SOCAT SST, back to the equilibrator temperature (i.e. back to the original measurement) using the Takahashi *et al*.^21^ temperature correction used by SOCAT. The *f*CO_2 (sw)_ consistent with the reference SST climate data record is then calculated using the Humphreys^22^ temperature correction. This process simply reverses part of the original calculation performed by SOCAT (using the metadata), before then inserting the SST reference data and repeating the forward calculation. The process and theory are explained in detail within Goddijn-Murphy *et al*.^6^ therefore here we focus on the main process, and updates from the previous work. For the transformation of *f*CO_2 (sw)_ between different temperatures we use PyCO2SYS (v1.8.3.3)^23,24^ because these tools are traceable and they enable the use of both the Takahashi *et al*.^21^ and updated Humphreys^22^ temperature sensitivities.

For the first step, the *f*CO_2 (sw)_ reported at SOCAT SST were converted back to the equilibrator temperature using the same linear temperature sensitivity^21^ (with respect to the natural logarithm *f*CO_2 (sw)_ and temperature) used within the SOCAT processing^2,25^. If the air pressure and equilibrator pressure are recorded these were provided to PyCO2SYS. If the equilibrator pressure was not provided (17% of the observations), we use the air pressure + 3 hPa due to the overpressure generally maintained in ships^26^, which is consistent with the SOCAT processing^25^. If the equilibrator temperature was not reported (12% of the observations), the reverse calculation step was omitted and the SOCAT SST was considered the equilibration temperature (e.g. a membrane-based system, in close proximity to and at the same temperature as the water inlet).

The second step transforms the *f*CO_2 (sw)_ within the equilibrator or near the membrane to the reference SST data (i.e., the climate data record). In this step the *f*CO_2 (sw)_ was transformed using the updated temperature sensitivity described in Humphreys^22^, based on equations governing the marine carbonate system and the van ‘t Hoff equation. The updated temperature sensitivity (of Humphreys^22^) generally indicates a smaller sensitivity at lower temperatures and a larger temperature sensitivity at higher temperatures in comparison to the quadratic Takahashi *et al*.^21^ approach. The differences occur due to the Humphreys^22^ approach using the OceanSODA complete carbonate system dataset^27^ to carry out synthetic Takahashi *et al*.^21^ experiments at each location (1° spatial resolution) to retrieve the b_h_ term required by the van ‘t Hoff equation. The b_h_ was then parameterised as a function of SST, SSS and *f*CO_2 (sw)_, where the fCO_2 (sw)_ acts as a proxy for the dissolved inorganic carbon (DIC) to total alkalinity (TA) ratio. Therefore the approach does not assume an isochemical change^28^. The assumption of isochemical conditions (i.e. using the Takahashi *et al*. approach) would have a small impact for small temperature adjustments (i.e <1 °C)^22,28^. However, the impact will increase for fCO_2 (sw)_ observations that require larger temperature adjustments (i.e >1 °C) or occur in waters with DIC/TA ratios that diverge from those used to estimate the Takahashi *et al*.^21^ approach. Thus the use of the updated temperature sensitivity allows the recalculation process to consider more complex interactions and improve the recalculated fCO_2 (sw)_^22,28^.

It is noted that combinations of SST, SSS and *f*CO_2 (sw)_ that do not occur regularly in the global oceans (as assessed using the OceanSODA data) may lead to more unconstrained b_h_, and therefore temperature sensitivities, which could be improved with regional fits of the coefficients used in Humphreys^22^. We do not see this as a limitation for our analysis on the global scale because this detail is only relevant for ~0.1% of the SOCAT database or ~40,000 data points (these are the data within SOCAT database that have low salinity of less than 20 and high *f*CO_2 (sw)_ conditions greater than 700 μatm and typically occur in coastal, estuarine conditions).

Equivalent partial pressure of CO_2_ (*p*CO_2 (sw)_) is also provided by converting each *f*CO_2 (sw)_ to *p*CO_2 (sw)_ at the reference temperature using PyCO2SYS and providing the air pressure, so that both variants of the CO_2_ measurements are provided. The SOCAT *f*CO_2 (sw)_ observations that are made prior to 1980 (i.e., before the start of the CCI-SST climate data record) are not recalculated as the reference SST cannot be provided. Therefore ~50,000 observations between 1957 and 1979 do not undergo the recalculation process but remain present in the synthesis files.

Finally, we provide the uncertainty on the recalculated *f*CO_2 (sw)_ and *p*CO_2 (sw)_ which considers the uncertainties in the SOCAT SST and atmospheric pressure from the SOCAT protocols^25^, and the reference temperature uncertainty. These were propagated through the recalculation process using PyCO2SYS.

### Gridding into a monthly 1° synthesis file

The original SOCAT and recalculated observations were then gridded at monthly 1° resolution following the SOCAT gridding methodology^29^ into both unweighted and cruise-weighted variants of the observations. The unweighted gridding considers all observations within a monthly 1° grid cell of equal weighting. The cruise-weighted gridding follows the SOCAT developed method^29^ and considers that not all individual cruises sample at the same frequency (i.e some sample every minute, whereas others could be every 20 minutes), therefore an unweighted monthly 1° grid cell would be weighted towards the value of those from the highest frequency sampling. The cruise-weighted gridding therefore considers each individual cruise within a monthly 1° grid cell first, and then averages the cruise means for each monthly 1° grid cell. Results from both gridding procedures are generated (i.e., unweighted, and cruise-weighted), and the reference SST are also gridded following the same procedures. Additionally, we provide equivalent gridded recalculated *p*CO_2 (sw)_ that some users may use (Table 1 summarises the netCDF variable name for these data).

**Table 1:** Summary of the netCDF variables within the gridded recalculated SOCAT dataset.

Variable	NetCDF Variable Name	Units
Reference temperature unweighted	sst_reference_unweighted	°C
Reference temperature cruise-weighted	sst_reference_weighted	°C
Recalculated *f*CO_2 (sw)_ unweighted	fco2_recalculated_ave_unwtd	μatm
Recalculated *f*CO_2 (sw)_ cruise-weighted	fco2_recalculated_ave_weighted	μatm
Recalculated *p*CO_2 (sw)_ unweighted	pco2_recalculated_ave_unwtd	μatm
Recalculated *p*CO_2 (sw)_ cruise-weighted	pco2_recalculated_ave_weighted	μatm

As a consistency check to confirm the gridding method and precision, values within the gridded dataset are cross-checked against the original SOCAT gridded dataset. The unweighted SOCAT *f*CO_2 (sw)_ showed a mean absolute difference of 0.04 μatm and for the cruise-weighted *f*CO_2 (sw)_ a difference of 0.22 μatm (N = 384,729). Within the unweighted data, ~1200 monthly 1° regions (~0.3%) have an absolute difference greater than 1 μatm, which occur in locations where SOCAT *f*CO_2 (sw)_ observations could not be matched to the satellite reference data and therefore were not included in the gridding. The cruise-weighted data has ~10,000 of the monthly 1° regions (~2%) with a absolute difference greater than 1 μatm, which occur in the same regions as the unweighted data.

## Data Record

The recalculated SOCATv2025 dataset is available at Ford *et al*.^30^. The dataset contains four files. The first file (a.tsv file) contains the individual observations from the original SOCAT dataset (i.e observations with an uncertainty less than 5 μatm) which has been appended with six new columns: the daily reference satellite SST (T_reference), the uncertainty on the daily reference satellite SST (T_reference_uncertainty), the *f*CO_2 (sw)_ (recalculated_fCO2) and *p*CO_2 (sw)_ at the reference temperature (recalculated_pCO2) and the uncertainty on the recalculated fCO_2 (sw)_ (recalculated_fCO2_uncertainty) and pCO_2 (sw)_ (recalculated_pCO2_uncertainty).

The second file (a netCDF file) contains the monthly 1° gridded dataset containing the original SOCAT gridded observations, with corresponding fields for the recalculated observations at the reference SST and associated reference SST observation as both cruise-weighted and unweighted variants (variable names are summarised in Table 1). We refer the reader to the netCDF metadata for further information on the specific fields, which includes minimum, maximum and standard deviation for each recalculated variables. The netCDF format is consistent with the original SOCAT gridded netCDF dataset.

The third and fourth files contains the SOCATv2025 Flag E dataset (i.e. observations with an uncertainty less than 10 uatm but greater than 5 μatm) and are provided as a.tsv file for the individual observations that make up the Flag E data, and the monthly 1° gridded dataset in netCDF format. These files have the same format as described for the first two datasets.

The recalculated SOCAT dataset will continue to be produced annually for all future releases of SOCAT as a community best effort and as funding allows. The repository information and datasets will be given on the FluxEngine^31,32^ GitHub repository which also contains the underlying analysis software and links to the previous versions of the recalculated data (https://github.com/oceanflux-ghg/FluxEngine).

## Technical Validation

### Assessing the warm bias in SOCAT SST

**Fig. 1:**
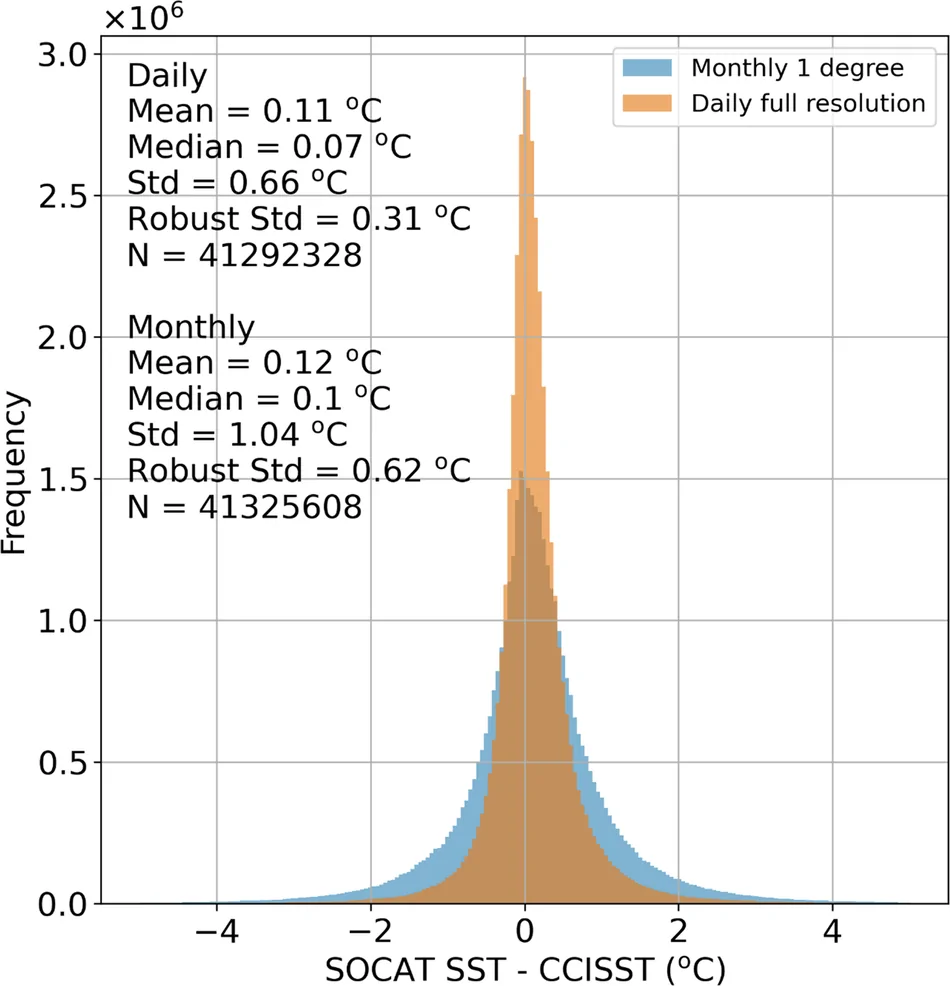
Histogram of the SOCAT SST minus the bias corrected CCI-SST plotted in 0.05 °C bins for the monthly 1°, and daily full resolution reference SST data. In plot statistics are mean bias (Mean), median bias (Median), standard deviation (Std), robust standard deviation (Robust Std) and number of observations (N).

As previously mentioned, the assessment of the warm bias within the SOCAT SST dataset was previously based on monthly 1° reference temperature data^13,14,16^. This inherently introduced a spatial and temporal mismatch between the SOCAT SST and reference temperature observations, and this mismatch could lead to an over- or underestimation of the warming bias. By using daily full spatial resolution reference temperature data the mismatch in time and space is reduced. This is apparent from the narrowing of the histogram when using the daily full resolution data in comparison to the monthly equivalent (Fig. 1). Using the daily CCI-SST data indicated a significant global mean of the SOCAT minus reference SST difference of 0.11 °C (Two-tailed t-test; t(41292327) = 1115; p < 0.01), compared to the equivalent analysis using the monthly 1° reference which identified a 0.12 °C mean bias (Fig. 1) across the period 1980 to 2024. This indicates that the warm bias within the SOCAT data is slightly overestimated by ~0.01 °C if it is characterised using monthly 1° SST data. The CCI-SST reference used to analyse the data within Fig. 1 has been first corrected for the cool bias with respect to SST drifters (see earlier methods). Therefore the warm bias would be increased by another 0.04 °C if this cool bias had not been removed from the CCI-SST reference dataset (i.e., the daily mean bias would appear to be 0.15 °C instead of 0.11 °C).

**Fig. 2:**
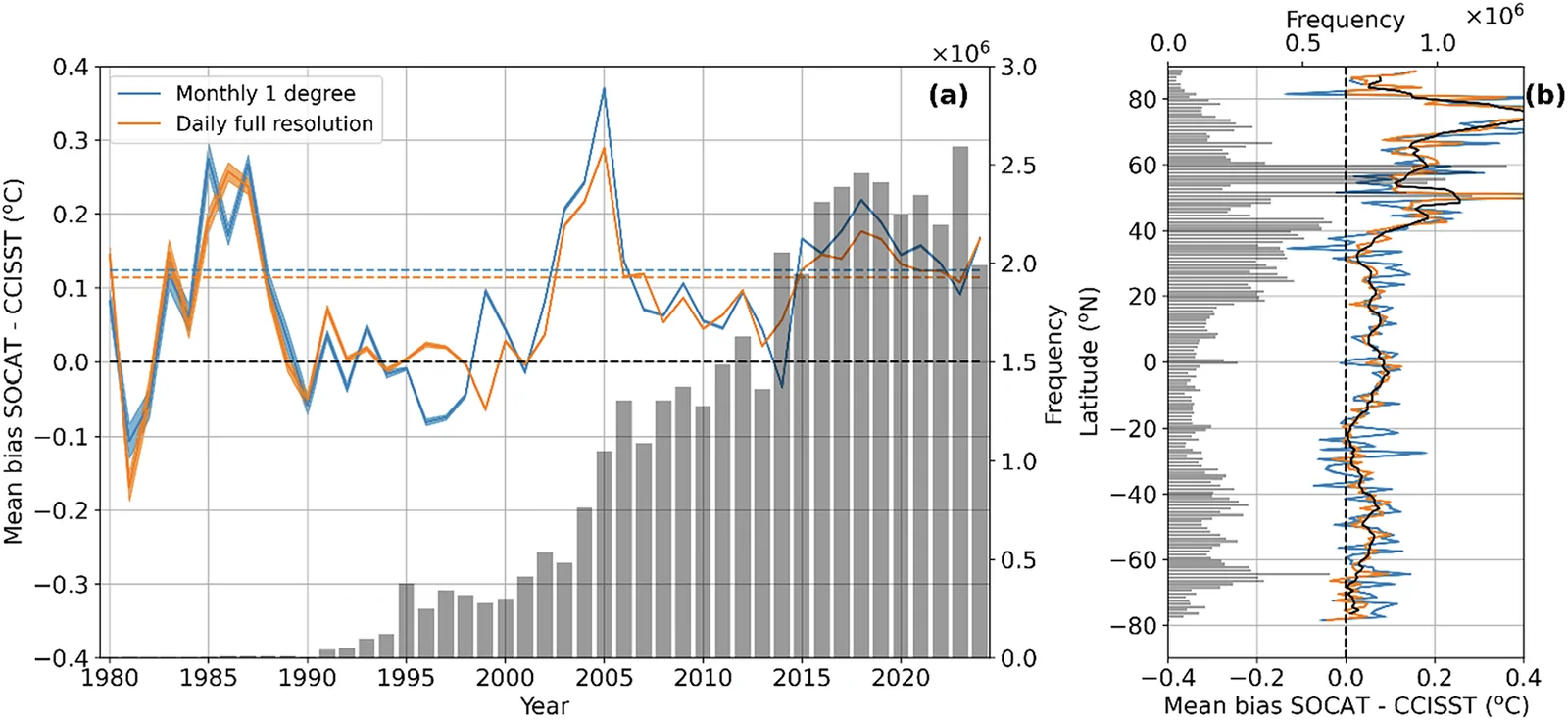
(**a**) Mean annual SOCAT SST minus the bias corrected CCI-SST between 1980 and 2024 (left y-axis). Blue line indicates the monthly 1° data, and orange line the daily full spatial resolution CCI-SST observations. Coloured banding represents the standard error on the calculated bias for the monthly 1° (blue) and daily full spatial resolution (orange). Dashed coloured lines indicate the global mean values from Fig. 1. Dashed black line indicates a bias of 0. Grey bars indicate the number of observations for each year (right y-axis). (**b**) Mean SOCAT SST minus bias corrected CCI-SST for 1° latitude bins considering all observations. Coloured lines are the same as in (a). Black solid line indicates a 5° moving mean of the daily full resolution (orange solid line).

To assess the temporal stability of the SOCAT warm bias, the mean warm bias was extracted for each year between 1980 and 2024 (Fig. 2a). All years, except 1989 and 1992, indicated significant differences between the SOCAT and reference SST (Two tailed t-test; p < .01).In the 1980s, a large warm bias of the order ~0.3 °C was identified but the number of SOCAT SST observations in this period was lower than later years (~60,000 between 1980 to 1990). From 1990, the number of SOCAT observations increases and the mean bias stabilises at around ~0.01 °C until 2000 where it reduced to near 0 °C. After 2000 the mean bias shows an increase from 0 °C in 2000 to ~0.1 °C in 2024, with a notably elevated bias in the 2003 to 2005 period. The analysis therefore indicates that the warm bias is not a temporally stable feature and appears to be increasing with time since ~2000. This analysis also reinforces that using monthly 1° reference SST data generally overestimates the warm bias in the SOCAT dataset, further supporting the use of the daily SST dataset.

Dong *et al*.^7^ indicated that there was a latitudinal variability to the SST warm bias within the monthly 1° gridded SOCAT SST data. Within our analysis, the warm bias shows a consistent latitudinal variability (Fig. 2b) except our analysis shows a stronger warm bias within the high latitude North Hemisphere.

**Fig. 3:**
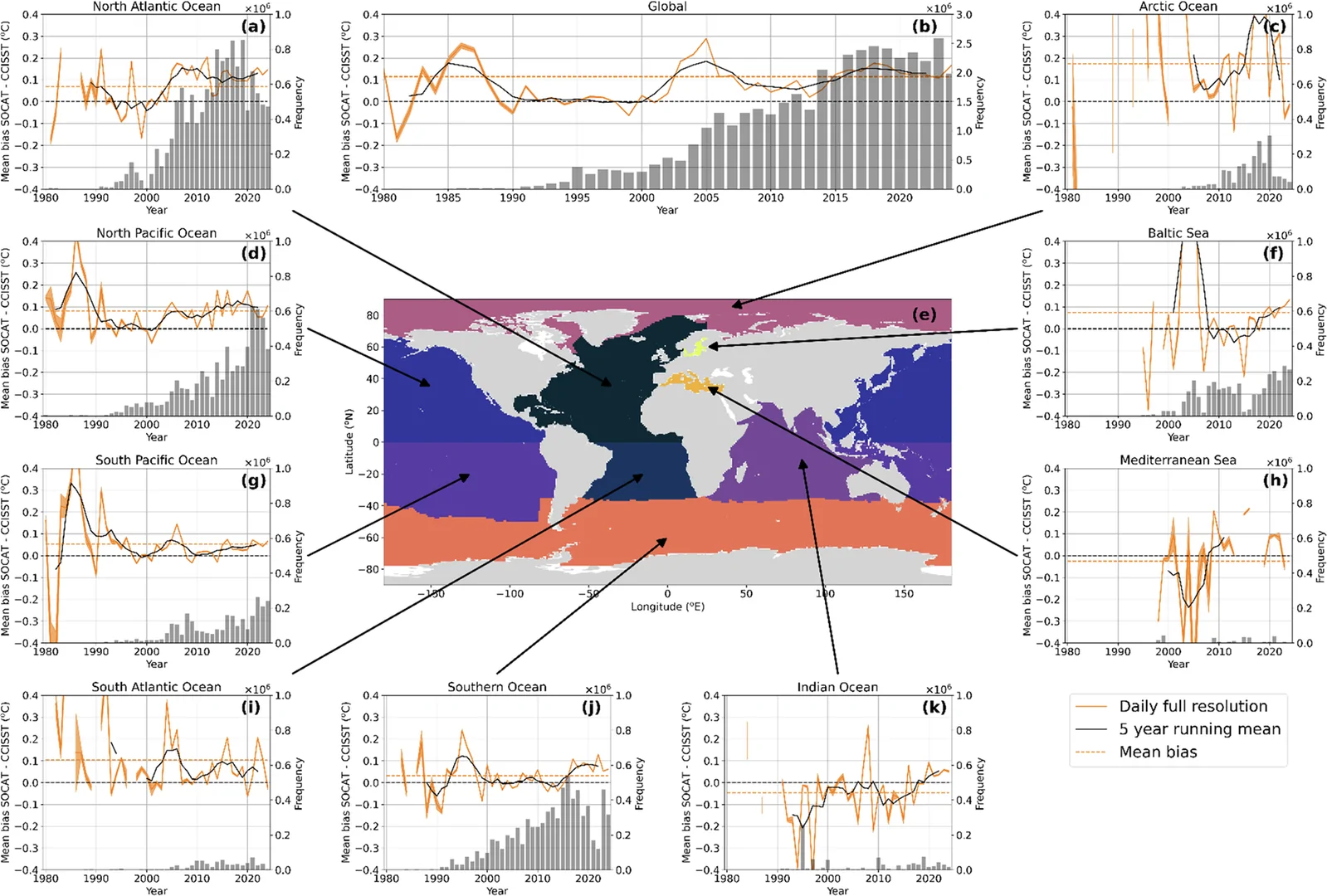
(**a**) Mean annual SOCAT SST minus the bias corrected CCI-SST between 1980 and 2024, for North Atlantic Ocean (left y-axis). Solid orange line indicates the daily full spatial resolution CCI-SST observations. Dashed coloured line indicate the mean bias for the region. Dashed black line indicates a bias of 0. Solid black line indicates a five year moving average (mean) of the daily full resolution mean bias. Grey bars indicate the number of observations for each year (right y-axis). Note y-axis limits in (**b**) are different from the remaining subplots. (**b**) same as (**a**) for all the data (as in Fig. 2). (**c**) same as **(a**) for the Arctic Ocean. (d) same as (a) for the North Pacific Ocean. (**e**) RECCAP region definitions. (**f**) same as (**a**) for Baltic Sea. (**g**) same as (**a**) for South Pacific Ocean. (**h**) same as (**a**) for Mediterranean Sea. (**i**) same as (**a**) for South Atlantic Ocean. (**j**) same as (**a**) for Southern Ocean. (**k**) same as (**a**) for Indian Ocean. Basemap in (**e**) from Natural Earth v4.0.0 (https://www.naturalearthdata.com/).

The variability of the warm bias was explored regionally for the recalculated dataset. Figure 3 shows the mean bias timeseries for the eight Regional Carbon Cycle Assessment and Processes (RECCAP2) regions (and additional regions including the Baltic Sea). These timeseries highlight some of the spatial variability and how regionally there are different temporal changes in the warm bias. These regional differences will reflect the individual systems and platforms used to collect the SOCAT SST data within each region and indicates which regions are likely driving the global mean warm bias. Within the North Atlantic and North Pacific Oceans the warm bias appears to increase from 2000 until present, with the North Atlantic generally having a larger bias than the North Pacific Ocean (Fig. 3a,d). The Southern Ocean tends to show a mean bias of ~0 °C for the period 2000 to 2010 around°, before rapidly increasing from 2015 to a maxima in the most recent years (Fig. 3j). The South Pacific Ocean generally showed a small mean bias around ~0.04 °C with fluctuations between years (Fig. 3g). The Indian Ocean, Arctic Ocean and Mediterranean Sea have limited data, and therefore the mean bias is likely sensitive to variations in the data density (Fig. 3c,f,k). There is a tendency for the Arctic Ocean to have large warm bias (Fig. 3c), and the Indian Ocean (Fig. 3k) to have a cool bias in the 1990 that increases to a warm bias by 2023. In the global analysis (Fig. 2) there was a notable increase in the warm bias in 2003 to 2005 period, which can be attributed to the Baltic Sea (Fig. 3f). These results indicate that there are geographical variabilities in the warm bias, but also temporal variability within each region (Fig. 3), which is different from the global analysis (Fig. 2). We note that the CCI-SST was corrected for a cool bias, that was assumed to be temporally and spatially homogenous (i.e., the fixed bias correction of 0.04 K covered within the methods), which may impact the regional analyses of the warm bias. However, Fig. 3 shows that many regions have a mean SOCAT SST to CCI-SST bias larger than the cool bias correction of 0.04 K, which are temporally varying suggesting that the fixed bias correction is not the source.

**Fig. 4:**
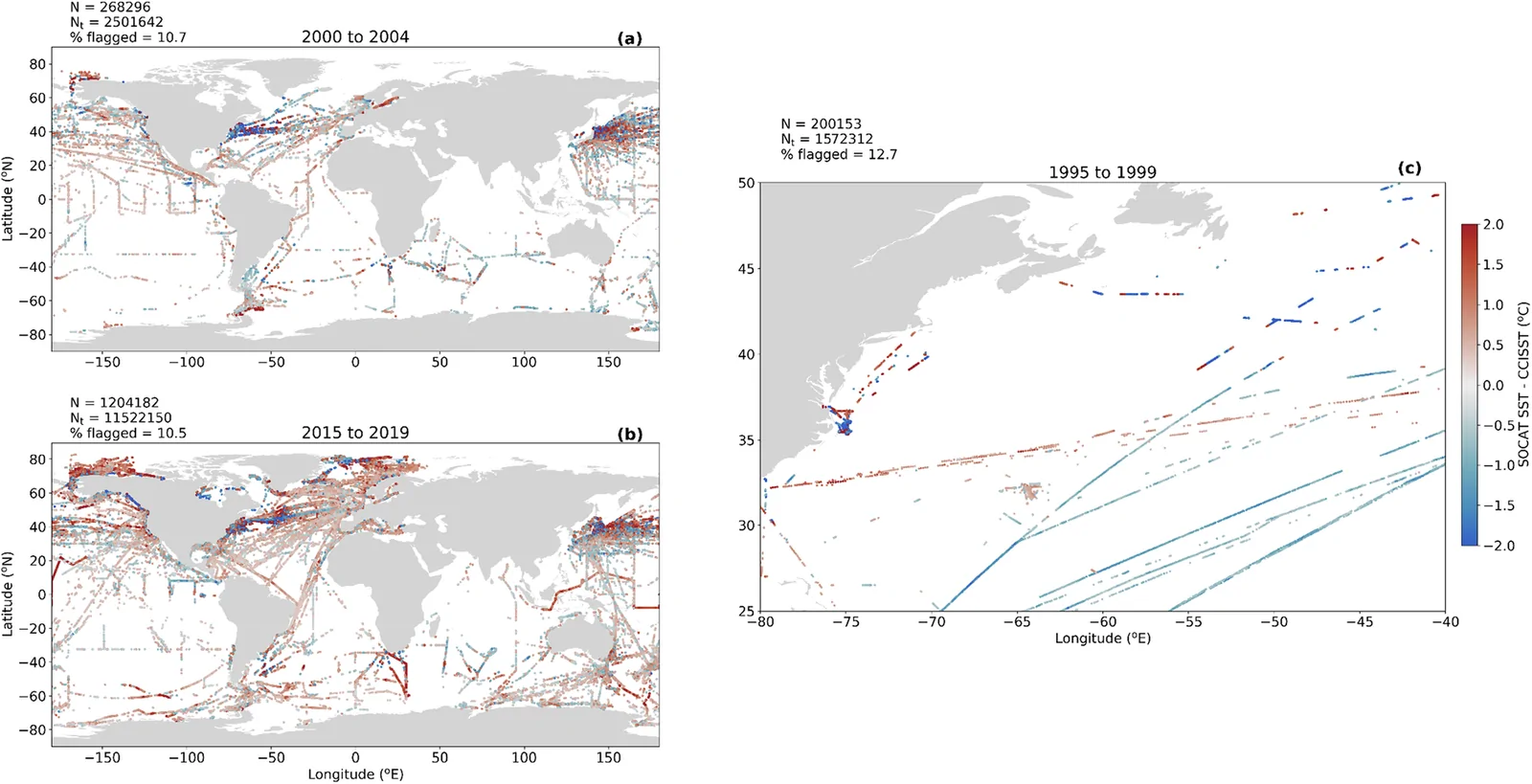
Global map of individual cruises where the SOCAT SST minus CCI-SST difference was greater than two times the CCI-SST uncertainty (~95% confidence), for cruises in the time period above each plot. Statistics above plot are number of flagged data (N), number of total SOCAT observations (N_t_) and percentage of data flagged (% flagged). Each subplot indicates a different period from (**a**) 2000 to 2004 and (**b**) 2015 to 2019. (**c**) shows a subset region over the Gulf Stream for the period 1995 to 1999. Basemap from Natural Earth v4.0.0 (https://www.naturalearthdata.com/).

The colocation between SOCAT SST and the CCI-SST reference SST allows for the recalculation of the SOCAT *f*CO_2 (sw)_. Equally the analysis can aid in identifying and understanding the underlying datapoints that contribute to the warm bias within the SOCAT dataset. For each SOCAT SST observation the SOCAT minus CCI-SST difference can be considered alongside the uncertainty information provided with the CCI-SST to indicate the significance of a particular bias. For example, Fig. 4 shows the individual SOCAT observations where this difference falls outside two times the CCI-SST uncertainty, which would indicate a ~95% confidence that the difference was significant, for two time periods. These difference results are binned into five year periods, which broadly indicates that about 10% of the SOCAT observations get flagged within the analysis, and individual cruises with particularly large differences are apparent (e.g., some with differences that are greater than 2 °C; Fig. 4b). This analysis could therefore provide another level of SOCAT quality control and would allow submitters to the SOCAT dataset to return and re-check their data (i.e., towards reducing these warm biases within the SOCAT dataset or identifying the possible causes). Equally, some biases may be real and can be ignored within the SOCAT quality control, for example within the vicinity of the Gulf Stream boundaries where small changes in sampling times between the *in situ* SOCAT data and the daily SST reference will likely, and correctly, lead to differences due to the rapidly shifting temperature boundaries and moving eddies (i.e Fig. 4c showing large positive and negative bias geographical on the same cruise line in the Gulf Stream region).

### Impact of updating the temperature sensitivity on the recalculated *f*CO_2 (sw)_

**Fig. 5:**
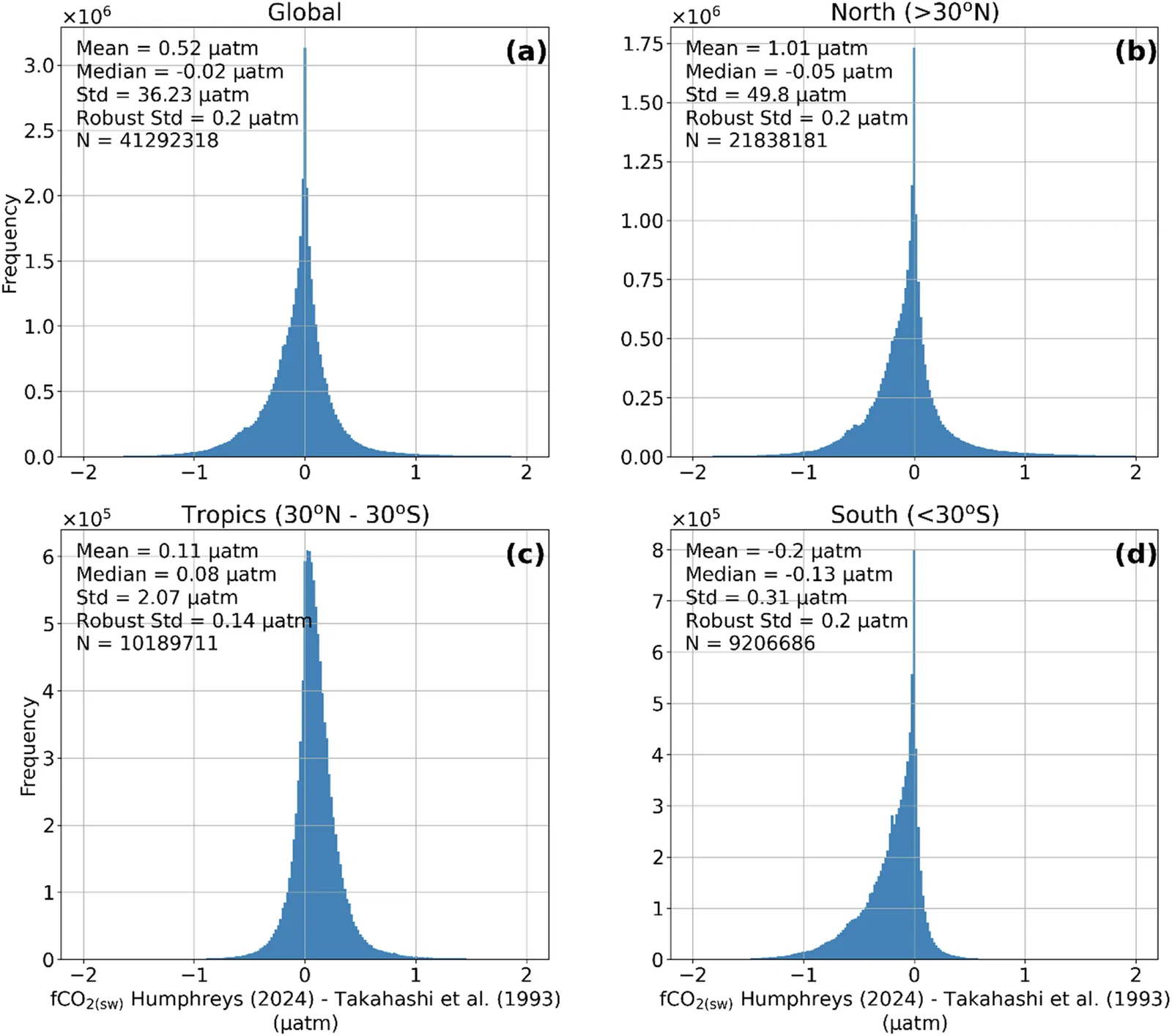
(**a**) Histogram of the global difference in the recalculated *f*CO_2 (sw)_ using the Humphreys^22^ and Takahashi *et al*.^21^ temperature sensitivities with the daily bias corrected CCI-SST. In plot statistics are mean bias (Mean), median bias (Median), standard deviation (Std), robust standard deviation (Robust Std) and number of observations (N). (**b**) same as (**a**) but for the North Hemisphere (>30°N). (**c**) same as (**a**) for the Tropics (30°N to 30°S). (**d**) same as (**a**) for the Southern Hemisphere (<30°S).

The choice of the temperature sensitivity approach used to recalculate the SOCAT reported *f*CO_2 (sw)_ to the satellite reference temperature will affect the resulting recalculated *f*CO_2 (sw)_. Comparing the updated temperature sensitivity of Humphreys^22^ to the previously used Takahashi *et al*.^21^ we find small differences in the recalculated *f*CO_2 (sw)_ (Fig. 5). On the global scale, a median difference of −0.02 μatm indicates that the Humphreys^22^ temperature sensitivity produces slightly lower *f*CO_2 (sw)_ (Fig. 5a). This global difference is due to the Humphreys^22^ approach having a lower sensitivity in cold waters with higher DIC/TA ratios^28^, and a higher sensitivity in warmer waters when compared to Takahashi *et al*.^21^. This feature is apparent when the differences were split regionally, into the Northern Hemisphere (Fig. 5b), Tropics (Fig. 5c) and Southern Hemisphere (Fig. 5d). For example, the Northern and Southern Hemispheres there is a tendency for the Humphreys^22^ method to produce lower *f*CO_2 (sw)_ shown by negative median biases of −0.05 μatm and −0.13 μatm, respectively. These regions largely cover the cooler waters, where the temperature sensitivity was smaller. The reciprocal is apparent in the Tropics (positive median bias) where the temperature sensitivity is larger than Takahashi *et al*.^21^ In some coastal locations, with low salinity and high *f*CO_2 (sw)_, the Humphreys^22^ temperature sensitivities can lead to larger *f*CO_2 (sw)_ differences. These are highlighted by the elevated standard deviations shown particularly in the Northern Hemisphere (Fig. 5b). Although these *f*CO_2 (sw)_ differences can be relatively large, they affect only < 0.1% of the SOCAT data analysed (or ~40,000 observations).

### Reasoning for using the CCI-SST climate data record instead of OI-SST data record

**Fig. 6:**
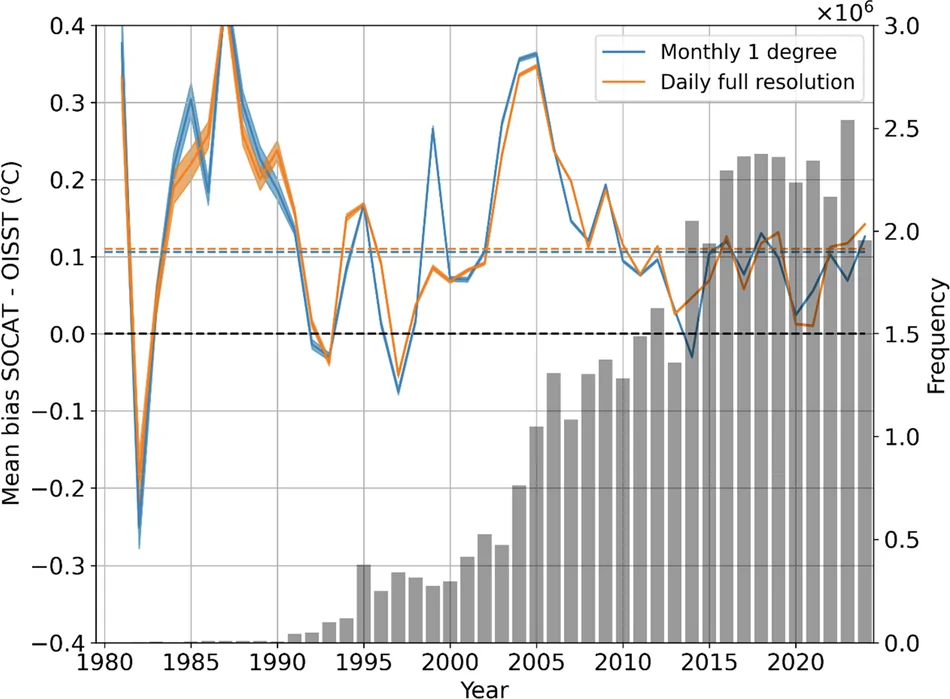
Mean annual SOCAT SST minus the OI-SSTv2.1 between 1981 and 2024 (left y-axis). Blue line indicates the monthly 1° data, and orange line the daily 0.25° spatial resolution OI-SST observations. Dashed coloured lines indicate the global mean values from Fig. 7. Dashed black line indicates a bias of 0. Grey bars indicate the number of observations for each year (right y-axis).

Some previous versions of the recalculated SOCAT datasets have included those recalculated using OI-SSTv2.1 or CCI-SSTv2.1^14,16^. The temporal stability and consistency of the reference SST data is important when assessing and correcting the warm bias within SOCAT dataset, as any drift or changes in the SST reference will affect the correction of the warm bias and therefore the resultant global ocean CO_2_ sink values. The OI-SSTv2.1 data have an inconsistency through time due to an updated methodology applied after 2015, as described in Huang *et al*.^9^, where data prior (1981 to 2014) follow the previous methodology (v2.0). An inconsistency in the generation of the SST reference data could manifest into a bias change with respect to SST drifters. The climate record intercomparison^19^ and an intercomparison in Yang *et al*.^33^ showed that for the period 1981 to 2010 the OI-SST was cold by about 0.1°C with respect to the *in situ* SST drifter observations, before transitioning towards being consistent to the SST drifters in 2022. This drift was apparent within the annual mean biases retrieved for OI-SSTv2.1, where larger and more variable warm biases between 1981 to 2010 were observed (Fig. 6) compared to CCI-SSTv3.0 (Fig. 2).

**Fig. 7:**
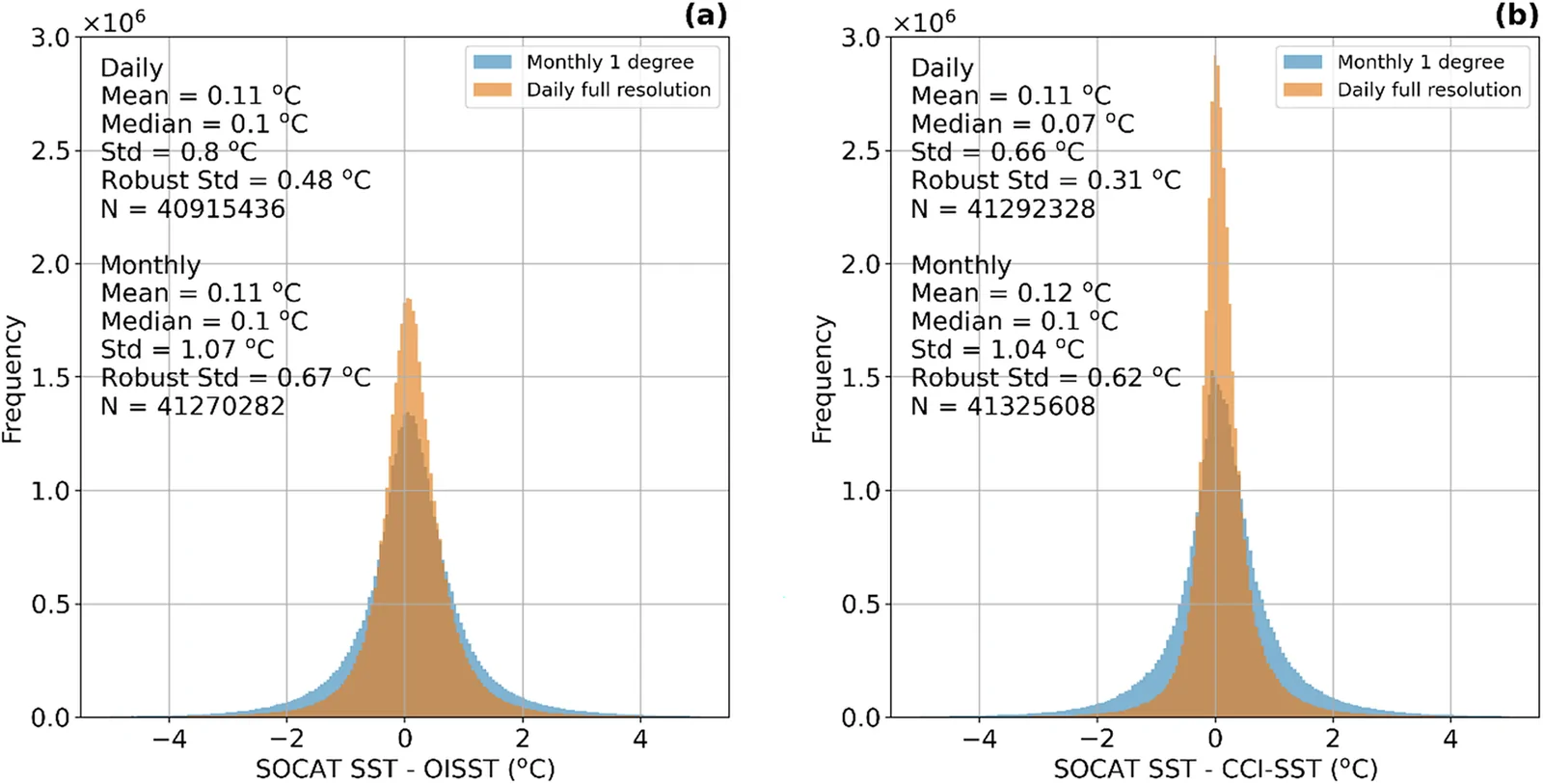
(**a**) Histogram of the SOCAT SST minus the bias corrected OI-SSTv2.1 plotted in 0.05 °C bins for the monthly 1°, and daily 0.25° resolution reference SST data. In plot statistics are mean bias (Mean), median bias (Median), standard deviation (Std), robust standard deviation (Robust Std) and number of observations (N). (**b**) same as (a) but for the CCI-SSTv3.0 as plotted in Fig. 1.

The OI-SST provides daily SST, but these have a reduced spatial resolution of 0.25°s (i.e., ~25 km at the equator) compared to the CCI-SST daily data which are ~5 km. If the same analysis conducted for the daily CCI-SST data (Fig. 1) is applied to the daily OI-SST data, there is a narrowing of the histogram (Fig. 7). But the narrowing is smaller than that achieved when using the daily CCI-SST data. This is particularly clear when evaluating the standard deviation (σ) and robust standard deviation (σ_r_; median absolute deviation scaled by 1.4826 so that it is consistent with the standard deviation for a normal distribution) of each distribution (Fig. 1; Table 2, where σ = 0.66 for CCI-SST versus σ = 0.80 for OI-SST, and σ_r_ = 0.31 for CCI-SST versus σ_r_ = 0.48 for OI-SST). This finding is consistent with the increased spatial resolution of the CCI-SST (i.e., 5 km with CCI-SST versus the 25 km of OI-SST) further reducing the spatial mismatch between the SOCAT observations and the reference SST dataset used to recalculate them. The reduction in the spatial mismatch will improve the representativeness of the recalculated SOCAT observations when considering individual cruise observations, or smaller regional subsets, further improvement in the applicability of these recalculated data.

**Table 2:** Summary statistics from the comparison of SOCAT SST minus the satellite reference SST at both monthly 1° and the daily native resolution of the reference SST dataset (CCI-SSTv3.0 = ~5 km; OI-SSTv2.1 = ~25 km).

Dataset	Monthly 1°				Daily full resolution			
	Mean (°C)	Median (°C)	Std, σ (°C)	Robust Std, σ_r_ (°C)	Mean (°C)	Median (°C)	Std, σ (°C)	Robust Std, σ_r_ (°C)
CCI-SSTv3.0	0.12	0.1	1.04	0.62	0.11	0.07	0.66	0.31
OI-SSTv2.1	0.11	0.1	1.07	0.67	0.11	0.1	0.80	0.48

The statistics are standard deviation (Std, σ) and robust standard deviation (Robust Std, σ_r_).

It should be noted that the gap filling techniques used by both the OI-SST and CCI-SST methodologies will smooth out features, such that the feature resolution will typically be coarser than the stated gridded resolution except when there is a high density of satellite observations^33,34^. The increased spatial mismatches due to the lower spatial resolution of the OI-SST data are consistent with the slightly larger mean bias that were identified in comparison to that identified by using the CCI-SST daily 5 km equivalent (Fig. 1; Table 2). The greater narrowing of the histogram for the CCI-SST (Fig. 1; Table 2) supports the conclusion that the higher spatial resolution of the CCI-SST is more appropriate for use with the SOCAT observations and enhances their use for studies in smaller regions or those that use individual cruises.

Overall the reduced temporal stability of the OI-SSTv2.1 throughout the 1981 to 2024 period and the higher spatial resolution of the CCI-SST which results in a closer temporal and spatial match to the SOCAT dataset observations, makes the CCI-SSTv3.0 more suitable for assessing and removing the warm bias within the SOCAT dataset.

Although we show that the CCI-SST is more suitable for assessing the warm bias within SOCAT, we must highlight that this is a particularly demanding application for any of these SST climate data records (i.e., requiring an accuracy and stability on the order of 0.01 °C). These climate data records are under constant development^9,10^, and this work highlights the importance of the ongoing work to revisit satellite-era data for the purpose of creating more accurate and stable climate data records.

### Demonstrating the impact of the recalculation on the estimate of the global ocean CO_2_ sink

**Fig. 8:**
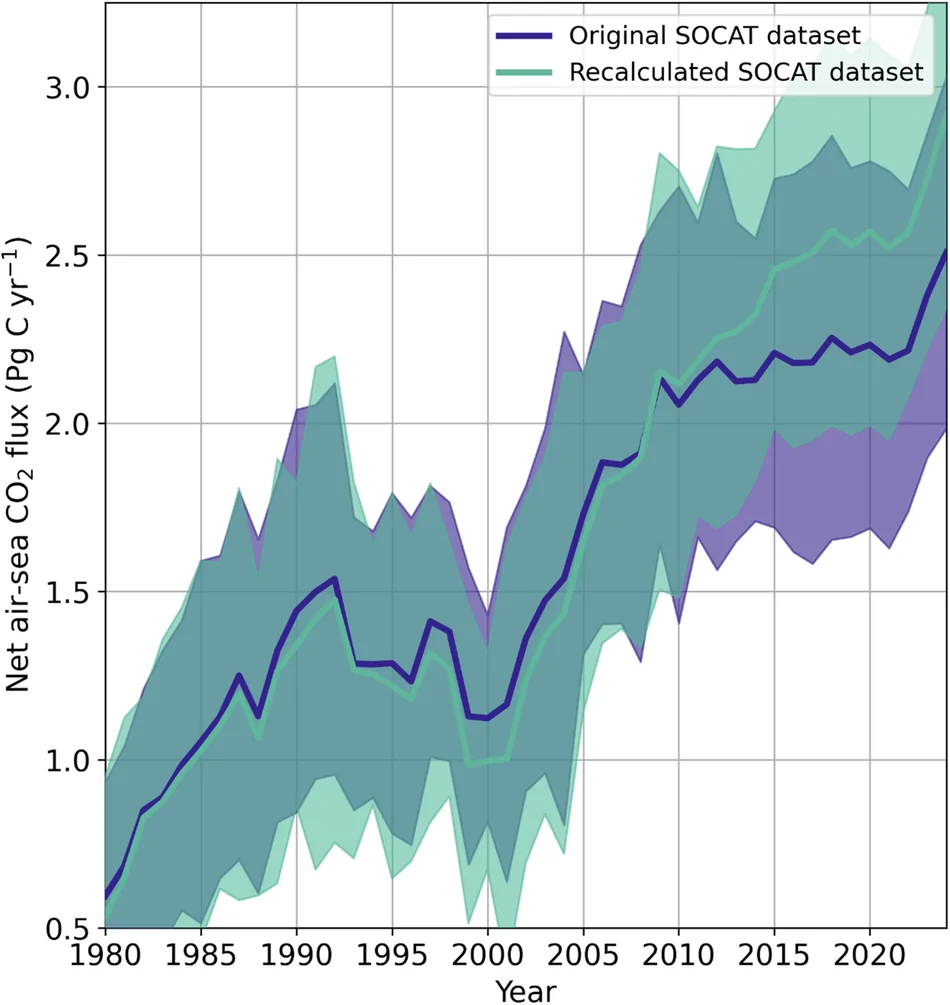
Global ocean net air-sea CO_2_ flux, or ocean CO_2_ sink using the UExP-FNN-U methodology. The ‘Original SOCAT dataset’ line indicates the UExP-FNN-U run with the original SOCAT dataset, and the ‘Recalculated SOCAT dataset’ line uses the recalculated SOCAT dataset as described within this paper. Positive net air-sea CO_2_ fluxes indicate CO_2_ uptake by the oceans. The ocean CO_2_ sink values presented do not include the fixed riverine flux adjustment as included within the Global Carbon Budget assessments. Shaded region indicates the 1σ uncertainty on the global ocean CO_2_ sink due only to the fCO_2 (sw)_ interpolation as described in Ford *et al*.^35^, as this is the component that would be directly affected by changing the original SOCAT to the recalculated SOCAT dataset.

The recalculation of the SOCAT *f*CO_2 (sw)_ to a reference SST dataset corrects for the warm bias present within SOCAT, which if left uncorrected, leads to a modified air-sea CO_2_ flux global ocean CO_2_ sink^7,8,35–37^. Here we use the University of Exeter Feed Forward Neural Network with uncertainties for interpolating the *f*CO_2 (sw)_ data and calculating the air-sea gas fluxes and net sink (UExP-FNN-U)^8,35^ to provide a single example of the impact of removing the warm bias (Fig. 8). The UExP-FNN-U methodology uses the self-organising map feed forward neural network (SOM-FNN) two step approach to estimate fCO_2 (sw)._ The SOM phase geographically clusters the global oceans into 16 regions with a similar seasonality in fCO_2 (sw)_. For each cluster region, an FNN ensemble is trained to estimate the fCO_2 (sw)_ within each region from a set of predictor variables. The approach follows similar principles to some other fCO_2 (sw)_ interpolation methodologies (e.g.^38,39^), but the different prediction approaches (e.g XG-Boost, decision trees, multi linear regression) and methodological structures could show different sensitivities, so this analysis is provided as an exemplar. Within the UExP-FNN-U methodology, the air-sea CO_2_ fluxes were computed following the bulk methods in Ford *et al*.^35^ but with the cool salty skin correction omitted.

The UExP-FNN-U methodology was first run using the original SOCAT *f*CO_2 (sw)_ observations (i.e. the non-recalculated version that contain the warm bias) to set the ‘baseline’, and this run is consistent with the majority of the current *f*CO_2 (sw)_ data-products used within the annual Global Carbon Budget assessments^1^. The UExP-FNN-U method was then run with the recalculated *f*CO_2 (sw)_ data that were recalculated using the daily bias corrected CCI-SST with the Humphrey’s temperature sensitivity (i.e., the dataset presented and described within this paper). The differences between the two analyses (Fig. 8) are generally consistent with the identified mean warm bias’s (i.e., those shown in Fig. 2). Around the year 2000 the recalculated data showed a ~0.05 Pg C yr^−1^ decrease in the ocean CO_2_ sink. After the year 2000, the difference grows to an increase in the ocean CO_2_ sink of ~0.4 Pg C yr^−1^ in 2024. These results show that if the original SOCAT dataset is used, the ocean CO_2_ uptake would likely be underestimated from 2010 onwards due to the increasing warm bias. This underlines the importance of recalculating the SOCAT data to a temporally stable climate data record before using them in any ocean carbon sink assessments.

## Data Availability

The recalculated SOCATv2025 dataset is available at Ford *et al*.^30^. The recalculated SOCAT dataset will continue to be produced and the repository information and datasets will be given on the FluxEngine^31,32^ GitHub repository (https://github.com/oceanflux-ghg/FluxEngine).

## Code availability

The FluxEngine Python toolbox (v4.2.0)^31,32^ contains the updated software implementation of the recalculation methodology. The main driver script for this is ‘fe_reanalyse_fco2_driver.py’, which can be run from the command line.
